# Draft Genome Sequence of *Salmonella enterica* subsp. *enterica* Serovar Orion Strain CRJJGF_00093 (Phylum *Gammaproteobacteria*)

**DOI:** 10.1128/genomeA.01063-16

**Published:** 2016-09-29

**Authors:** Sushim K. Gupta, Elizabeth A. McMillan, Charlene R. Jackson, Prerak T. Desai, Steffen Porwollik, Michael McClelland, Lari M. Hiott, Shaheen B. Humayoun, Jonathan G. Frye

**Affiliations:** a Bacterial Epidemiology and Antimicrobial Resistance Research Unit, USDA-ARS, Athens, Georgia, USA; b Department of Microbiology, University of Georgia, Athens, Georgia, USA; c Department of Microbiology and Molecular Genetics, University of California Irvine, Irvine, California, USA

## Abstract

Here, we report a 4.70-Mbp draft genome sequence of *Salmonella enterica* subsp. *enterica* serovar Orion strain CRJJGF_00093, isolated from a dog in 2005.

## GENOME ANNOUNCEMENT

*Salmonella enterica* subsp. *enterica* serovar Orion belongs to low-invasive groups of *Salmonella* pathogens (1), though *S*. Orion has been isolated from a variety of hosts, including humans (2, 3), cattle (4), ducks (5), and birds (6), across the globe. Here, we announce the draft genome sequence of *S*. Orion, isolated from a dog in 2005.

The *Salmonella* strain was isolated from a sick dog by NVSL (National Veterinary Services Laboratory) using standard microbiology techniques. The isolates were serotyped using *Salmonella* multiplex assay for rapid typing PCR (7), and a serotype antigenic formula was predicted using reads with SeqSero (8), which predicted the antigenic formula of 3,10:y:1,5, designated Orion. Using pulsed-field gel electrophoresis (PFGE), as described by PulseNet, the isolate displayed the PFGE pattern XLAX01.0009.ARS (9). Susceptibility testing for the strain was performed using broth microdilution plates for the Sensititre semiautomated antimicrobial susceptibility system (TREK Diagnostic Systems, Inc, Westlake, OH, USA), and guidelines of the Clinical and Laboratory Standards Institute (CLSI) were followed to interpret the results (10).

Genomic DNA was isolated from an overnight culture using the GenElute bacterial genomic DNA kit (Sigma-Aldrich, St. Louis, MO, USA). DNA libraries were constructed using the Nextera-XT DNA preparation kit, and paired-end sequencing was performed on the Illumina HiSeq 2500 platform (Illumina Inc., San Diego, CA, USA) using a 500-cycle MiSeq reagent kit. A total of 4,183,624 reads were generated. Reads were *de novo* assembled using Velvet (11), which assembled to 178 contigs ≥200 bp with an 83-fold average coverage. The combined length of the contigs was 4,701,421 bases with a G+C content of 52.19% and an *N*_50_ value of 58.9 kb. The contigs were ordered with Mauve (12) using the *Salmonella* LT2 genome sequences as reference; coding sequences and tRNAs were predicted with Prodigal (13) and ARAGORN (14), respectively. A total of 4,445 coding sequences (≥50 amino acids) and 43 tRNAs were predicted within the genome. Signal-peptide, CRISPR regions and prophages were predicted using Signalp (15), CRISPRFinder (16), and PHAST (17), respectively. We identified signal peptides in 414 coding sequences, two CRISPR loci, and three intact phages (Salmon_SP_004 [NC021774], Stx2_converting_1717 [NC011357], and Mannhe_YB_MnM_3927AP2 [NC028766]) in the analyzed contigs. The strain was susceptible to all the tested antibiotics, though we detected a cryptic aminoglycoside resistance gene, *aac6*-Iy, with ARG-ANNOT (18). The genome data generated from *S*. Orion can be helpful to understand the variations in genomic features that make *S*. Orion a member of a low-invasive group of *Salmonella* pathogens.

### Accession number(s).

The genome sequence of *Salmonella enterica* subsp. *enterica* serovar Orion strain CRJJGF_00093 has been deposited in the GenBank database (NCBI) under the accession number JQVX00000000. This paper describes the first version of the genome, JQVX01000000.
